# Balconies’ visual preferences: The case of residential apartments in Tehran, Iran

**DOI:** 10.1371/journal.pone.0324874

**Published:** 2025-07-29

**Authors:** Paria Akbari, Seyed-Abbas Yazdanfar, Saeid Norouzian-Maleki, Seyed-Bagher Hosseini

**Affiliations:** 1 School of Architecture and Environmental Design, Iran University of Science and Technology, Tehran, Iran; 2 Faculty of Architecture and Urban Planning, Shahid Beheshti University, Tehran, Iran; Islamic Azad University Mashhad Branch, IRAN, ISLAMIC REPUBLIC OF

## Abstract

Balcony as a border between internal and external plays a crucial role in determining the quality of the apartments. The current condition of apartment balconies in Tehran, indicates that balconies have been gradually been forgotten due to their non-functional features for users. In architecture, space quality is measured by user preferences and can serve as a guide for designers in future designs. The objective of the present study was to identify the dominant physical characteristics of balconies in Tehran and improve people’s visual preference from both outside and inside viewpoints. Photographing, Delphi technique and collecting public questionnaire were used to do the research, and quantitative data were gathered through administering questionnaires. Valid responses were gathered, then the collected data were analyzed using SPSS. According to the results, there is a significant difference between the two categories of pleasant and unpleasant. In addition, the characteristics of desirable images from the outside and inside are not always the same. From the outside view of the balcony, the area is the most effective parameter, which in pleasant images was between 50% and 75% of the façade area. Fully solid and completely fences parapet, were preferred from the outside view, while from the user’s point of view, the space from the inside of the balcony with a completely fences parapet was unpleasant and a completely solid or a combination of a solid and fences was preferred.

## 1. Introduction

### 1.1 Intermediate spaces

It is common for architecture to separate the space into interior and exterior areas [1], but the inside and outside space are not completely separable and there is always a connection between them that depends on the type of openings and the relationship between the walls [2]. Creation of the separating border and connection between them is the oldest architectural concept [3].

Architectural spaces that cannot be accurately classified as inside or outside and have characteristics of both categories and generally placed between them are considered as intermediary spaces. Japanese architect, Kurokawa, proposes the term “Grey Space” [4], Aldo van Eyck, the Dutch architect used the term “Twin phenomenon” [5], and Hermann Hertzberger used “Threshold” about these spaces [6].

Intermediate spaces act as borders in a more intricate manner, combining the attributes of two overlapping areas rather than just separating inside and outside spaces [7]. Visual comfort [8], thermal comfort [9], acoustic comfort of transitional spaces, gradation of the spatial layers in architectural design [1], and space analysis of intermediate zones studied in research. However, instead of relying on technology to design a housing envelope, designing intermediate spaces can offer ideas for modern house architecture and enhance its habitability [10].

Transitional spaces are both quantitative and qualitative concepts of spatial continuity [11]. Bonyani [12] has examined spatial continuity in both physical and mental domains and has identified terraces and balconies to be areas of physical continuity. These spaces, such as house entrances and courtyards, support social activities within the community, and contribute to the preservation of social unity and identity [13].

Previous studies have explored the function of the balcony space as a thermal, acoustic, and visual filter, as well as its role in aesthetic preferences, but the study of the physical characteristics of the balcony and its simultaneous function from the inside, the perspective of the space user, and from the outside, the perspective of those who observe building façades, has not been conducted. Achieving a balance between practical function and formal appearance in apartment balconies can enhance the designer’s capability to create a sustainable and usable design. This research focuses on addressing the research gap.

### 1.2 Balcony

Balconies as a type of intermediary space between open and closed areas, provide a link between private and public, as well as indoor and outdoor spaces; and function as a connection point between two separate zones [14]. Balcony, as a zone between internal and external influences on the residential space, primarily controls the noise, wind, excessive heat and dust [15]. Blocking direct summer sunlight, discomforting glare and undesirable harmful rays, providing luminous comfort [16], natural ventilation, reducing energy consumption [17–19], and noise protection [20] have been investigated in studies. But, the study focuses on the psychological aspect of balconies, believing that it should be an intermediary space that visually connects the different areas of outside and inside while preserving its original physical attributes [21]. A lot of people use the balcony to connect with others, be part of a bigger scene, and perceive social life [22].

The relationship between built-environment variables including distance from the city center, population density, housing prices, number of bus stops, as well as the neighborhood amenity and COVID-19 has been examined in research at different scales [23,24]. Furthermore, the COVID-19 pandemic has brought attention to the widespread desire for private outdoor spaces in houses. During the worldwide quarantine, numerous news and articles highlighted the increasing use and appreciation of balconies, as well as their integration into house design for welfare of society [25–27]. During the lockdown, Italian residents took to their balconies to socialize with their neighbors [28]. According to a study in Milan, students who have moderate to severe depressive symptoms are significantly more likely to reside in small apartments with unusable balconies [29]. During the lockdown in Belgium, an assessment of adult mental health revealed that participants who had a garden or balcony in their living space had lower GHQ scores, which indicated better mental health [30]. The pandemic has heightened the importance of balconies in apartments. To accommodate the necessary equipment for daily activities, the balcony needs to be large enough [31]. A study conducted in Tehran during this period revealed that balconies measuring more than 5 square meters had a significant impact on reducing GHQ scores and improving mental health [27]. Moreover, another study in Tehran on people’s housing preferences during this period indicated that the highest priority was given to the presence of a terrace and its design in determining housing selection priorities in terms of space [26].

Today’s, designing multi-family housing in terms of achieving comfort presents a challenge for city planners and architects, as it should be close to the principles of designing individual homes. In this regard, residents and experts agree that high quality of private open spaces is a significant factor to achieve this goal. A lot of people who buy or rent a new house, never accept the flats without open/semi-space. It indicates that private open space plays a crucial role in determining the quality of the apartment and residential environment [15,32].

### 1.3 Visual preferences

Individuals are inextricably linked to the environment. The interaction between humans and the built environment is important for creating knowledge in design disciplines. Knowledge gained from human reactions to the built environment can be effective in achieving better design decisions and creating a more harmonious interaction between humans and the built environment [33]. In this regard, for designers, planners, and sociologists, residential preferences are important, and studies are increasingly interested in analysis of the residential preferences, satisfaction, and choices [34–39]. Preferences reveal a variety of tendencies to satisfy human wants [40]. There are underlying motives in every preference and choice that enable a person to select one among the options accessible for him/her [41]. Human preferences are the result of how they perceive their environment through their senses and their experiences, beliefs, values, social and economic status, and future expectations [42].

Human perception, including visual and other senses, can impact how the environment is assessed [43]. While all senses play a role in identifying the relationship between humans and their environment, sight and visual appearance play a major role. It is of great importance for describing and assessing personal evaluations of architectural scenes to create desirable places to meet the sensory-emotional needs of people [44].

People show varied preferences towards specific environmental phenomena. Key factors influencing environmental preference include: 1) physical features such as the shape, size, and height; 2) vegetation, and the presence of greenery; and 3) human interest [45]. The impact of cultural differences is observed as an essential element in determining preferences [46,47]. Varied cultural identities impact how individuals experience their environments [48]. Research on environmental preferences has mainly focused on natural or rural environments, with significantly less emphasis on urban environments [49,50]. Curvature and sharpness of architectural façades [51], color, ornaments, materials, roof, openings, shape and proportions [52], surface complexity, silhouette complexity and façade articulation [53], and visual preference have assessed in studies.

This study intends to investigate the physical characteristics of residential balconies and their relationship with people’s visual preferences. Findings of this study provide recommendations and suggestions for designers and architects, which consider the double role of the balcony with the interior space and exterior environment, and provide satisfaction to the residents on both sides.

## 2. Methodology

Considering the main purpose of the research, it consists of four main phases: 1) the survey of the current state of balconies, 2) identification of parameters through the Delphi technique, 3) assessment of the people’s visual preferences, and 4) data analysis.

### 2.1 First phase

To study the current situation, by photographing the balconies of the apartments, the physical characteristics based on the significant differences between them are selected as the primary parameters of the research.

In this research, residential apartments in Tehran have been selected, because the distribution of residential apartment units in Tehran is about 81%, which is the largest in the country. To choose a homogeneous population, we have limited the geographical area in Tehran based on social and cultural factors to reduce the intervening factors as much as possible. Kalantari and Taslimi Tehrani (2013) analyzed and identified the different lifestyles in Tehran and also determined the geographical distribution of them. Lifestyles in Tehran are classified into three categories: upper class, middle class, and lower class, and the middle class is divided into traditional and modern categories.

**Fig 1:**
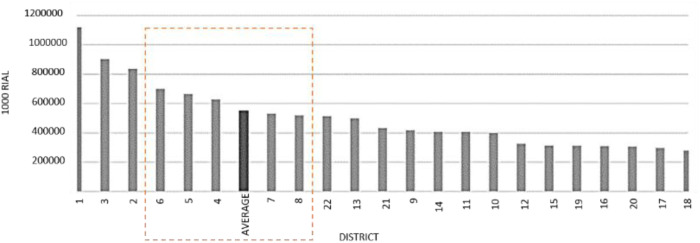
Average price of residential apartments in Tehran.

A study on Tehran’s lifestyles conducted by the Institute of Social Studies measured financial issues such as food, clothing, housing, and other items such as social interactions and art. The study concluded that despite the heterogeneity of respondents, uniformity prevails over differentiation in Tehran’s lifestyles. The predominance of homogeneity on the differentiation of the middle class of society has been proven in research. Thus, the modern middle class, whose similarity in lifestyle is more evident, was chosen (districts one to eight). To select a homogeneous society, in addition to the lifestyle, the housing price is also an effective factor in choosing the research area. According to the information on the Iranian Statistics Center on January 2022, district 1 has the highest price and district 18 has the lowest price among the 22 districts of Tehran. Fig 1 shows the weighted average price of residential apartments in Tehran. From an economic point of view, out of eight regions of middle class lifestyle, regions 4, 5, 6, 7, and 8 have been selected as the economic middle class.

Apartment façades were photographed for a three-month period in this study, and images of the south and north blocks were captured with location recording. In the photographs, attempts were made to ensure that the distance from the façade and the angle of the photographs were identical and from the front of the façade.

### 2.2 Second phase

In this study, the Delphi technique was used to select more important parameters of the research. The technique used where there is a limited knowledge of a particular issue [54,55]. It is widely used to collect data from a small group of experts in a particular field to reach a consensus on a particular issue in the real world [56–58]. If the group is homogeneous, a smaller sample size of ten to fifteen provide sufficient results. The panel members remain unknown to each other and their interaction is managed [25,59].

Delphi technique has involved a series of rounds and questionnaires. The outcomes of every round are assessed by participants in subsequent phases after reviewing the feedback from the other Delphi experts [60]. Multiple studies indicate that the number of rounds is influenced by the research objective. Most studies recommend that the Delphi process lasting two or three rounds is adequate to achieve consensus [54,61]. After each round, the feedbacks are analyzed, and a new questionnaire is sent to panel members for the next round. This process continues until a predetermined criterion is met [62,63].

**Round 1:** The Delphi technique typically begins with an open-ended to collect data from the experts [64], and then convert the responses to a structured questionnaire for the second round [65].

**Round 2:** Participants are given the second questionnaire and requested to evaluate the items and score them to determine their priorities. Disagreement and agreement are typically found at the conclusion of this round [66,67].

**Round 3:** Every participant is given a questionnaire containing the summarized items from the earlier round and is requested to reassess or finalize their evaluations [68]. This provides panel members with the opportunity to clarify their data and decisions. Nevertheless, there is only a slight difference from the previous round [69–71].

Choosing participants is an essential phase in the Delphi technique. The survey is conducted with qualified participants who have relevant background, expertise, or experience related to the topic. They should be able to engage in various stages and reassess earlier judgments until a consensus is achieved [72].

In this study, a pilot study was conducted with five experts to evaluate the clarity of the subject and the questions during Delphi rounds. Using Delphi technique, experts confirmed the structure and design. The input gathered from the pilot study was incorporated into the final version of the Delphi questionnaires.

The experts were invited from university professors of the Tehran College of Fine Arts, Shahid Beheshti University Faculty of Architecture and Urban Planning, and IUST’s School of Architecture and Environmental Design who teach in the field of architectural and urban design and are active in civil and urban projects. An email letter was sent to them that introduced the study and the Delphi process, which was included in the invitation.

### 2.3 Third phase

Visual evaluation techniques were utilized in conducting this study. The primary approach used in the study is the visual questionnaire technique, which involves visuals for assessing the user’s perception of the space.

Questionnaires provide tools for collecting data about people’s knowledge, beliefs, behavior, and attitudes [73]. Questionnaires must align with research goals, and it should be clear from the beginning how the findings will be used. They provide the possibility of collecting quantitative data in a standardized way so that the data are consistent for analysis [74].

This research did not involve any human experimentation or private data and did not require ethical approval. The respondents completed the questionnaire in August, 2024, and all the participants were adults who were informed of the purpose of the study and who voluntarily agreed to participate in the survey. They signed a written statement agreeing to the use of the opinions and data they provided in the study. Moreover, all the data were collected and analyzed in strict compliance with relevant laws and regulations. Formal confirmation was obtained from the Academic Committee of the School of Architecture and Environmental Design, Iran University of Science and Technology.

In terms of methodology, the use of photographs was chosen for preference studies. Software has enabled the manipulation of photos and specific factors, allowing for the examination of the impact of a particular element within a controlled simulated environment. Many scholars favor using computer-based environmental simulations [75], and the suitability of simulations for environmental research has been widely discussed [76–78].

In this phase of the study, people are asked to complete a questionnaire where they are shown simulated images of balconies from both inside and outside. A 5 point Likert scale was used to determine the visual preferences of balconies, with 5 being the highest and 1 the lowest. The images were modeled according to the research variables in the Autodesk Revit software, and confounding variables such as perspective and material were controlled during the modeling process. The photo-questionnaire inquired about age, gender, ownership, education, income level, and the number of family members in a confidential manner without revealing the subjects’ names.

### 2.4 Fourth phase

**Fig 2:**
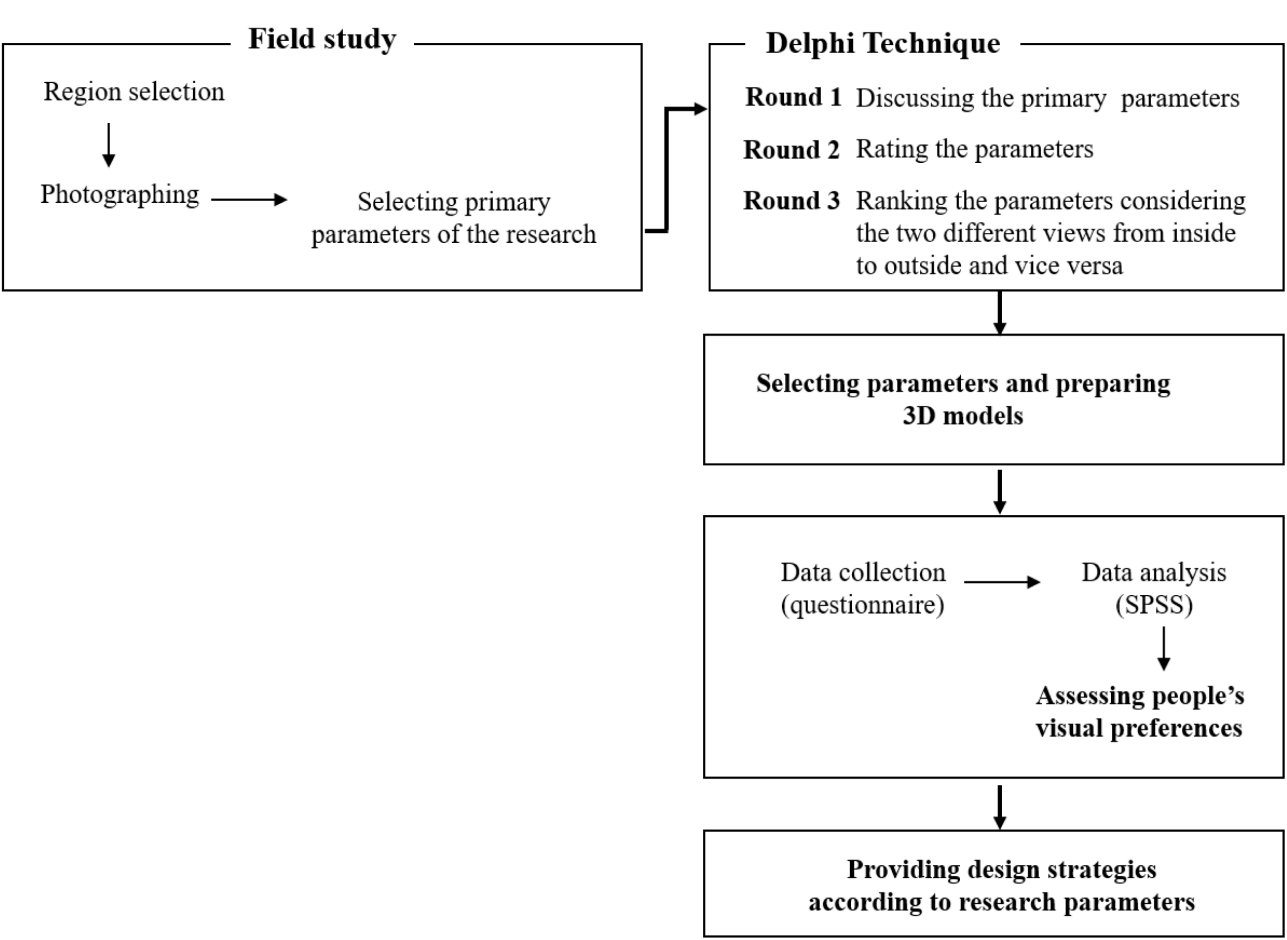
Research process.

SPSS version 22.0 for Windows was used to analyze the data from the questionnaire. The primary focus was on the descriptive analysis of the participants’ responses, particularly the means and medians of the data. To compare the differences between different groups of images, T-test analysis was employed (Fig 2).

## 3. Findings

In the first phase, to extract the most important physical features of the balcony in residential apartments, the façades of four-, five- and six-story residential apartments in districts 4, 5, 6, 7, and 8 were randomly photographed, and 1024 photographs of the northern and southern façades were taken in total in all the areas.

In the first step of the Delphi technique, interviews were subsequently conducted with 17 University experts to comment on the chosen parameters. The primary parameters selected from the photos of the 5 regions were as follows: 1) location, 2) area of the balcony in the façade, 3) geometry, 4) placement type in the building mass, and 5) material. The experts were asked to suggest new ones or omit someone. According to the results, parapets and the number of balconies in the façade were added by the professors, the material was suggested as a controlled parameter, and the three parameters of greenness, audience distance, and landscape were also recommended as controlled parameters.

In the second step of Delphi, the experts were asked to rate the importance of the six parameters of the first round of the Delphi survey from 1 to 5 (1 = very low importance, and 5 = very high importance). Among the 28 questionnaires sent, twenty specialists completed the questionnaires within two weeks. The results extracted from the responses of architecture professors are indicated in Table 1.

**Table 1:** Primary parameters.

	Parameter	Mean
1	Numbers of balcony	3.20
2	Geometry	4.30
3	Location	4.60
4	Area (Area of the balcony in the façade)	4.40
5	Placement (Type of placement in the building mass)	4.20
6	Parapet	3.80

According to the results, the parameters of the number of balconies are lower than the average mean in terms of importance, and five parameters with an average higher than 3.40 were chosen for the third Delphi round.

**Fig 3:**
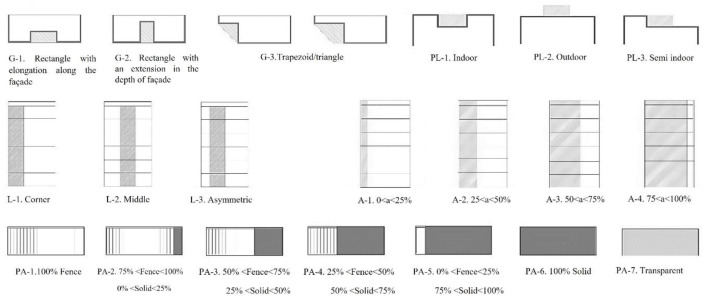
Parameters and diagrams of outside.

**Fig 4:**
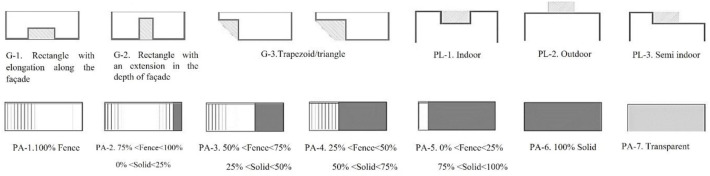
Parameters and diagrams of inside.

In addition to the main parameters, categories related to the main parameters have been extracted from the photos, and experts have been asked to rate the parameters from 1 to 5 (1 = very low importance, and 5 = very high importance). Considering the two different views from inside to outside and vice versa, the parameters are divided into two categories according to the type of view. Figs 3 and 4 depict the parameters of the view from the outside and inside along with the corresponding diagrams.

Among the 25 questionnaires sent, twenty experts completed the questionnaires. The parameters that have an average higher than 3.40 were selected for further research (Tables 2 and 3).

**Table 2:** Mean score of outside parameters.

Main parameter	Mean	Parameters	Mean
Geometry	3.80	Rectangle with elongation along the façade	4.30
		Rectangle with an extension in the depth	2.40
		Trapezoid/triangle	3.20
Location	3.90	Corner	3.80
		Middle	3.70
		Asymmetric	2.50
Placement	4.0	Indoor	4.10
		Outdoor	2.30
		Semi indoor	3.60
Parapet	3.70	100% Fence	3.70
		75% < Fence<100% 0% < Solid<25%	3.90
		50% < Fence<75% 25% < Solid<50%	3.00
		25% < Fence<50% 50% < Solid<75%	2.70
		0% < Fence<25% 75% < Solid<100%	3.60
		100% Solid	3.90
		Transparent	2.60
Area	**4.0**	0 < a < 25%	2.20
		25 < a < 50%	3.80
		50 < a < 75%	3.60
		75 < a < 100%	2.80

**Table 3:** Mean scores of inside parameters.

Main parameter	Mean	Parameter	Mean
Geometry	3.60	Rectangle with elongation along the façade	4.20
		Rectangle with an extension in the depth	2.00
		Trapezoid/triangle	3.60
Placement	4.50	Indoor	4.10
		Outdoor	3.20
		Semi indoor	3.80
Parapet	3.70	100% Fence	4.10
		75% < Fence<100% 0% < Solid<25%	3.60
		50% < Fence<75% 25% < Solid<50%	4.00
		25% < Fence<50% 50% < Solid<75%	4.10
		0% < Fence<25% 75% < Solid<100%	3.80
		100% Solid	4.00
		Transparent	2.70

**Fig 5:**
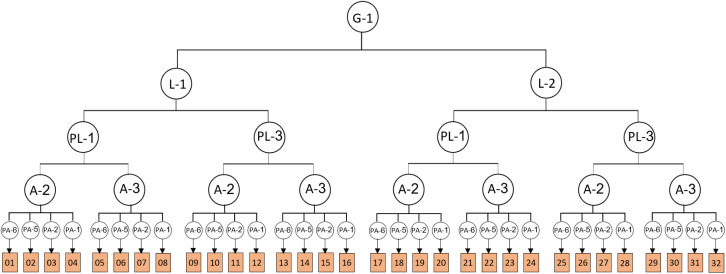
Parameters of outside 3D modeling.

**Fig 6:**
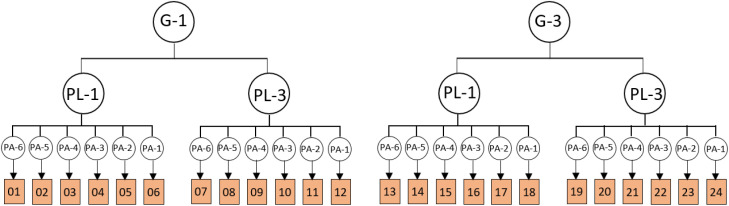
Parameters of inside 3D modeling.

Parameters with an average higher-than-average value were selected. To model images of balconies, a combination of selected parameters is used, and parameters such as materials, greenness, audience distance, and background are considered fixed parameters. Figs 5 and 6 present the process of combining parameters for 3D modeling. According to the diagram, a total of 32 images were prepared for viewing from the outside and were named 1–32. Additionally, 24 images were provided and labeled from 1 to 24 for viewing from inside to outside.

**Fig 7:**
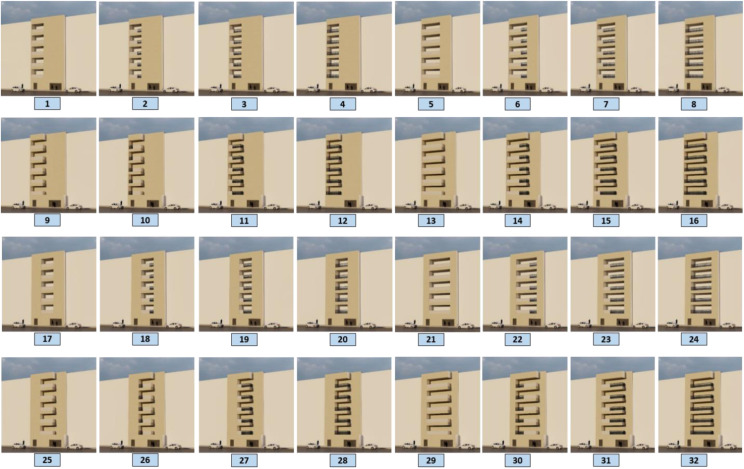
Outside 3D modeling.

**Fig 8:**
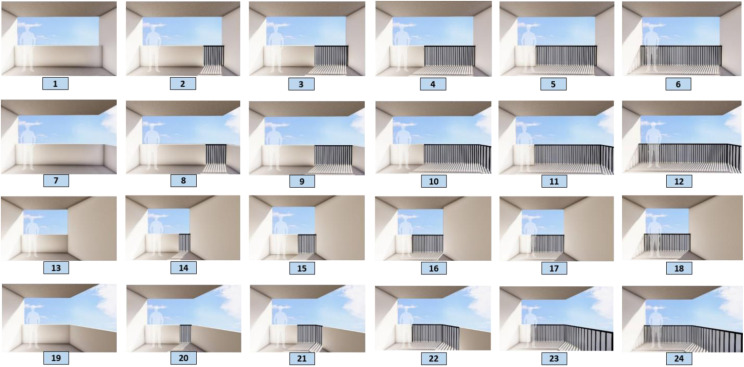
Inside 3D modeling.

The experimental questionnaire consisted of 66 items in 3 sections, including demographic data (10 items), residential images of the outdoor view of the balcony (32 items), and images of the indoor scene of the balcony (24 items), which were sent to people to assess their visual preferences. People are asked to rate their visual preferences of the images from 1 to 5 (1 = very unpleasant, and 5 = very pleasant). Figs 7 and 8 illustrate the 3D models used in the research.

We used a questionnaire to collect the data. Residents of Tehran were selected for this study. Over one month in August 2024, 362 valid responses were received. According to Tables 4 and 5, the average age of the participants was 33.9 years. The size of the household in this research is 3.1 people, the average time of people’s presence in their current house is 9.2 years, and the average area of the balconies is 3.2 square meters.

**Table 4:** Descriptive statistics of the personal data of the participants.

	*Age*	*Residence period*	*Balcony* *Area*	*Number of family members*
Mean	33.9	9.2	3.2	3.1
N	362	362	362	362
Std. Deviation	8.32	8.39	2.68	1.07

**Table 5:** Socio-Demographic data of the respondents.

		Frequency	Percent
Gender	Female	237	65.50
	Male	125	34.50
Education	Lower than diploma degree	4	1.10
	Diploma	12	3.30
	Bachelor degree	122	33.70
	Master’s degree and Ph.D.	224	61.90
Ownership	Owner	253	69.90
	Tenant	109	30.10
Monthly income (in Iranian Rial)	i < 100,000,000	5	1.40
	100,000,000 < i < 150,000,000	57	15.70
	150,000,000 < i < 250,000,000	114	31.50
	i > 250,000,000	186	51.40

A total of 34.5% of the respondents were men, 65.5% were women, approximately 70% of the participants were homeowners, and 30% were tenants. In terms of education, most of the respondents were educated, and more than 61% of the respondents had master’s and doctorate degrees.

According to the respondents’ ratings of the internal and external images based on the 5-point Likert scale, which is used to measure survey participants’ opinions, attitudes, and motivations, the respondents’ frequency and average scores are shown in Tables 6 and 7.

**Table 6:** Average scores of outside images.

Image	N	Frequency				Mean	Std. Deviation
		1	2	4	5		
O01	362	129	208	23	02	1.787	0.785
O02		166	184	11	0	1.610	0.674
O03		28	121	179	134	3.193	1.217
O04		22	122	133	85	3.378	1.322
O05		03	89	145	125	3.829	1.174
O06		11	158	163	30	3.119	1.153
O07		12	135	172	43	3.273	1.177
O08		15	67	126	154	3.931	1.240
O09		107	233	19	03	1.834	0.744
O10		83	207	69	03	2.177	1.019
O11		51	181	120	10	2.605	1.163
O12		25	111	116	110	3.483	1.375
O13		05	81	132	144	3.909	1.189
O14		08	118	202	34	3.376	1.100
O15		03	97	213	49	3.575	1.051
O16		0	34	113	215	4.406	0.898
O17		191	153	16	02	1.577	0.760
O18		226	135	01	0	1.381	0.503
O19		146	199	17	0	1.691	0.705
O20		26	151	155	30	3.033	1.211
O21		02	97	186	77	3.660	1.106
O22		08	194	147	13	2.898	1.080
O23		21	148	170	23	3.072	1.168
O24		03	56	177	126	4.014	1.022
O25		53	219	82	08	2.373	1.056
O26		63	230	67	02	2.213	0.960
O27		34	140	196	19	2.997	1.201
O28		38	138	140	46	3.050	1.303
O29		02	84	161	115	3.837	1.128
O30		05	131	173	53	3.381	1.157
O31		07	93	196	66	3.610	1.112
O32		07	62	111	182	4.102	1.164

**Table 7:** Average scores of inside images.

Image	N	Frequency				Mean	Std. Deviation
		1	2	4	5		
I01	362	23	133	180	26	3.146	1.178
I02		50	178	124	10	2.630	1.168
I03		0	49	239	74	3.934	0.862
I04		0	72	198	92	3.856	1.016
I05		19	115	169	59	3.370	1.230
I06		29	116	146	71	3.315	1.317
I07		01	10	175	176	4.423	0.666
I08		05	102	201	54	3.544	1.094
I09		01	46	218	97	4.006	0.896
I10		02	38	218	104	4.061	0.869
I11		32	133	114	83	3.229	1.380
I12		44	118	106	94	3.243	1.446
I13		247	115	0	0	1.318	0.466
I14		218	144	0	0	1.398	0.490
I15		197	164	01	0	1.461	0.516
I16		113	239	10	0	1.743	0.598
I17		90	200	61	11	2.180	1.080
I18		94	191	57	20	2.221	1.158
I19		0	26	183	153	4.279	0.796
I20		55	219	67	21	2.392	1.124
I21		0	132	192	38	3.376	1.085
I22		0	72	186	104	3.889	1.036
I23		23	155	106	78	3.168	1.347
I24		43	115	98	106	3.301	1.466

**Fig 9:**
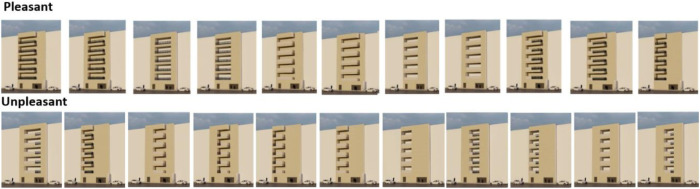
Pleasant and unpleasant outdoor images.

**Fig 10:**
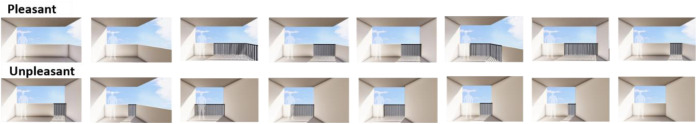
Pleasant and unpleasant indoor images.

Among the 32 outside images, 11 images with an average of more than average were chosen as pleasant images that were preferred by the people. In contrast, 11 images with low ratings were selected as the unpleasant category, and among the 24 internal images, 8 images with an average of more than 3.40 were selected as pleasant images, whereas the 8 images with the lowest scores were selected as unpleasant images. In the following, a comparison is made between these categories. Figs 9 and 10 show the images of the mentioned categories.

A t-test of the two categories of positive and negative preferences for internal and external images revealed a significant difference between them (Tables 8 and 9).

**Table 8:** Independent samples test of outside images.

	Levene’s Test for Equality of Variances		T-test for Equality of Means						
	F	Sig.	t	df	Sig. (2-tailed)	Mean Difference	Std. Error Difference	95% Confidence Interval of the Difference	
								Lower	Upper
Equal variances assumed	5.562	.029	11.160	20	.000	1.837	0.165	1.494	2.181
Equal variances not assumed			11.160	15.729	.000	1.837	0.165	1.488	2.187

**Table 9:** Independent samples test of inside images.

	Levene’s Test for Equality of Variances		T-test for Equality of Means						
	F	Sig.	t	df	Sig. (2-tailed)	Mean Difference	Std. Error Difference	95% Confidence Interval of the Difference	
								Lower	Upper
Equal variances assumed	7.709	.015	10.343	14	.000	2.081	0.201	1.650	2.513
Equal variances not assumed			10.343	10.709	.000	2.081	0.201	1.637	2.525

### 3.1 Comparison of pleasant and unpleasant images from the outside

In terms of location, both positions in the middle and in the corner (L-1 and L-2) were pleasant and unpleasant, and in terms of placement, both situations in the pictures (PL-1 and PL-3) were observed in two categories, pleasant and unpleasant. Therefore, it seems that these two variables were not among the determining features from the people’s point of view.

In terms of area, all the images selected in the pleased category, which shows people’s preference, have an area between 50% and 75% of the façade surface (A-3), and all the images with a smaller area of the balcony are placed in the opposite category, which shows that this variable is an effective variable considered by the people. The comparison of images according to the parapet variable indicates that the selected images in the pleased category had three types of parapet:

1- All fences (PA-1)
2- All solid (PA-6)
3- 75% <Fence<100%, and 0% < Solid<25% (PA-2).

The parapets of the first 8 images out of 11 pleasant images were fully solid or completely fence, which shows that, from the point of view of looking out to the balcony as part of the façade, the simplicity and lack of variety in the parapet was more favorable for people, and if they accept a combination of solid and fence parts for parapet, they prefer more fences.

In the unpleasant images, both categories of middle and corner locations and both types of placement can be observed, which indicates the neutrality of this variable in the unpleasantness of the images, as shown in the pleasant images. However, all the balconies in the unpleasant category have fewer balcony surfaces on the façade. With respect to the parapets of unpleasant images, three types have been undesirable:

1- 75% < Fence<100%, and 0% < Solid<25% (PA-2),
2- 0% < Fence<25%, and 75% < Solid<100% (PA-5),
3- 100% Solid (PA-6).

It highlights that solid parapets and the combination of solid parapets and more fences were selected in the unpleasant images as well as in the pleasant images, but balconies with full fences were not understood at all in the unpleasant images.

**Fig 11:**
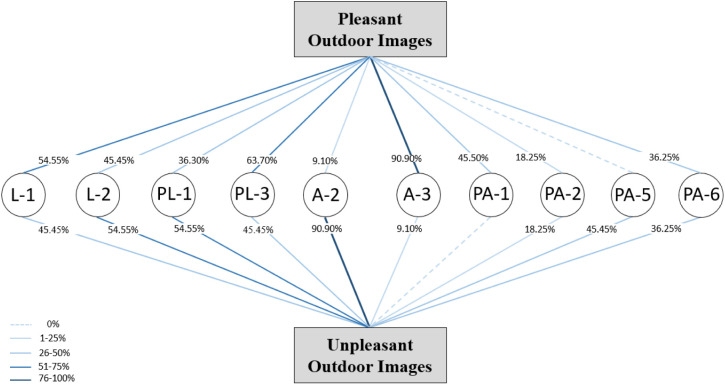
Pleasant and unpleasant outdoor images and relationships to the parameters.

Fig 11 shows the pleasant and unpleasant images and their relationships with the parameters. The diagram shows that there are no significant differences between the percentage of pleasant and unpleasant images with the parameters of location and placement, whereas there is a significant difference in the area. Approximately 90% of the pleasant images covered an area between 50% and 75% of the façade surface, and 90% of the unpleasant images covered an area between 25% and 50% of the façade surface. With respect to the parapet, 0% < fence<25% and 75% < solid<100% were not perceived in any of the pleasant images, and no fence types were observed in any of the unpleasant images.

### 3.2 Comparison of pleasant and unpleasant images from the inside

Most of the images preferred by the space user from the inside have rectangular geometry (G-1), two open sides (PL-3) and balconies with trapezoidal geometry (G-3), and one open side (PL-1) is less preferred. Therefore, it appears that these two variables are significant features from the people’s point of view in choosing balconies as pleasant or unpleasant. The comparison of images according to the parapet parameter demonstrated that the selected images in the pleased category had four types of parapet:

1- 50% < Fence<75%, and 25% < Solid<50% (PA-3)
2- 25% < Fence<50%, and 50% < Solid<75% (PA-4),
3- 0% < Fence<25%, and 75% < Solid<100% (PA-5),
4- 100% Solid (PA-6).

Considering the presence of a combination of solid and fences in the preferred images, it seems that there is no dominant preference for this combination from the users’ perspective, but the pleasantness of balconies with a completely solid parapet and the fact that balconies with all fences are not preferred in any way, it can be concluded that the users of this space prefer not to be completely visible from the outside.

Also, it can be referred that the rectangular geometry, two open sides, all solid parapets and combination of solid and fences for parapets were more desirable from the inside view. All six models of parapets were observed in the unpleasant category. This indicates that solids, fences and different combinations of these two factors have less of an impact on unpleasantness, and this depends on other parameters.

**Fig 12:**
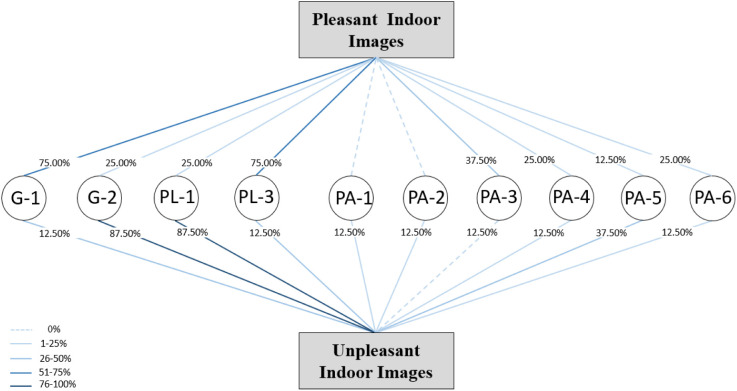
Pleasant and unpleasant indoor images and relationships to the parameters.

Almost 90% of the unpleasant images have trapezoidal geometry and an open side, which, according to the comparison of this result with the findings of the pleasant pictures, indicates that the geometry and the number of open sides from the point of view of the users in the indoor space are among the effective parameters. Fig 12 illustrates the pleasant and unpleasant images and their relationships with the parameters.

### 3.3 Comparison of pleasant/unpleasant images from the outside and inside

The first four images of the pleased category from the out have full fence parapet, and the next four images have solid parapets; however, from the perspective of the user on the balcony, the first two images in the pleased category have solid parapet, and the rest of the images have combined parapet of fences and solid parts such that these combinations are often unpleasant from the outside view of the balcony. These results confirm the difference in people’s preferences as observers of the balcony and users of this space.

From the outside, it was preferred to have a parapet that is completely solid and fenced. These are perceived as two types that are completely contradictory. Such a choice from the outside audience of the building can be considered as a simple parapet with less variety to enhance the visual comfort of the façade, as the combination of fenced and solid parts from the viewpoint of outside observers causes visual confusion and more variety in the façade. Some types do not properly separate the boundaries of the semi-open space on the façade.

**Fig 13:**
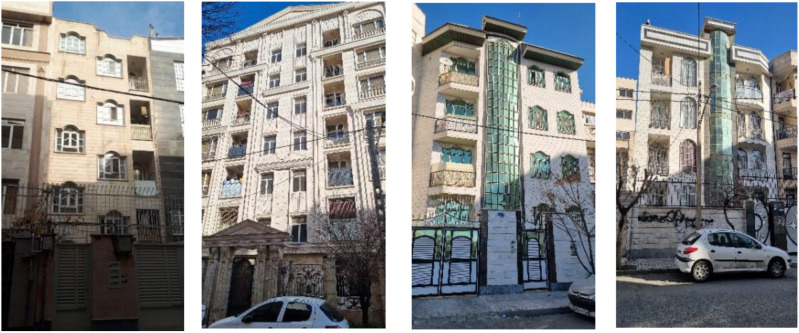
Balconies of apartments (Source: Authors).

The full parapet of the fence is not observed in any of the eight pleasant images from the inside view, whereas in the first four pleasant images from the outside, the parapet is the fence. The result of this difference can be understood in the photos taken in the first phase of this research. The photos reveal that some balconies with railings have been changed from the inside by the users of this space. Users have altered all or part of the parapets into solid parapets and limited the view from the outside (Fig 13).

A study in Tehran found that more than 80 percent of apartment residents do not make effective use of the available semi-open space due to the lack of privacy and awareness of the surroundings [79]. The use of railings or transparent materials in balconies is a cause of dissatisfaction in a study conducted in the Zafaranieh neighborhood of Tehran [80].

The covering of balconies can extend to the point where they are completely enclosed and removed entirely. Jangyu, Korea was surveyed to find out that residential apartments were being renovated by removing balconies to enlarge the size of the small bedrooms, create a new small bedroom, and expand the living area. The reduction of heating efficiency, sound insulation, and buffer space between indoor and outdoor spaces have been observed in some of these houses after the changes [81].

To put it another way, one of the most significant issues is the transition of residents from their private spaces to public spaces. It is undeniable that violating boundaries and privacy results in tension, discomfort and anxiety, while also destroying individual independence and respect [82]. Another study from Tehran has indicated that paying attention to the privacy and the beauty of the balcony in combination with other elements of the façade from a perceptual-semantic perspective has been effective on the satisfaction and preferences of residents. Residents have pointed to privacy, incorrect architectural space design, and unusability as reasons for not using the balcony [83].

In crowded or culturally traditional urban environments, balconies have a significant impact on emotional and psychological reactions. For women in traditional cultures where visual privacy is culturally expected, significant exposure may elicit sensations of vulnerability, anxiety, or social discomfort [84]. However, when balconies offer visual enclosure through design features such as lattice screens, greenery, or drapes, users indicate greater feelings of comfort, and emotional safety. The psychological desire for control over visibility is bolstered by these factors, which is closely associated with feelings of safety and autonomy [85]. Frequently, more enclosed balconies are perceived as extensions of indoor spaces, incentivizing activities such as reading, relaxing, or socializing in a semi-sheltered atmosphere [86–88]. Therefore, privacy-related design elements not only respond to social norms but also have a significant impact on how people emotionally connect with their home surroundings.

The comparison of the placement of the semi-open space of the balcony in the building mass from the outside view, which causes the number of different open sides in the balcony from the view of the user in the space, also demonstrates the desirability of this parameter, which differs from the view of the observer of the façade and the user inside the balcony. In relation to this parameter from the outside and as part of the building volume in both models, it could be argued: inside the building mass or part of it is outside the volume, it is acceptable, whereas from the user’s point of view from inside, the existence of two open sides has a positive effect on their pleasantness and preference. The results are in accordance with previous research on the openness of open and closed spaces, and this study also observed a similar outcome in the semi-open space area. Studies have examined the openness and closure of open spaces and closed ones, taking into account variables such as height, boundary height, physical and visual accessibility [89,90], and solid walls [91]. A study on the number of open-sides of semi-open spaces conducted in Hong Kong found that residents preferred the less enclosed open spaces, suggesting that openness may be a key design feature for open spaces in high-density [92]. Also, a study of closed rooms revealed that people experienced a more pleasant feeling with more open space on the walls [93].

## 4. Conclusion

The quality of architecture that humans experience every day has always been important for them. Among the human senses, the visual experience of space strongly influences desirability and preference. Accordingly, visual preferences are an important factor for identifying people’s architectural preferences. The role of these preferences is to identify what is desirable and undesirable for people.

Balconies are architectural spaces that affect preferences and visual amenities. Considering the location of this space as a border between inside and outside, in this study, the effects of this space and its characteristics from the inside and outside perspectives of the individual as an observer and user were examined.

The results revealed that there was a significant difference between the two pleasant and unpleasant categories and inside and outside images. According to the results from the outside view of the balconies, location and placement were not among the determining features, but the area was an effective parameter considered by the people. They preferred balconies with an area between 50% and 75% of the façade surface (A-3), fully solid or completely fence parapets, or a combination of solid and fence parts with more barriers.

According to the findings from the inside view of balconies, most of the images preferred by the space user have a rectangular geometry and two open sides. Considering the presence of a combination of solid and fences in the preferred images, it appears that there is no dominant preference for this combination from the users’ perspective.

These results can be useful for building designers because they can help identify effective visual differences in the desirability of the balcony. It is recommended that architects and designers pay attention to the difference between the two balcony views from the outside and inside, and choose the optimal mode that is preferred by the outside audience and the inside user.

Choosing a rectangular geometry, placing it in a building mass with two open sides, and combining a solid and fence parapet can simultaneously satisfy the desires of people from the inside and provide visual pleasure from the outside. In addition, the role of the balcony from the exterior view should be considered a component of the facade, whereas from the inside, it is considered a life space.

However, considering that the lack of desirability of these spaces from the inside causes changes by the users and further damages in the architects’ design for the urban appearance, it seems that the desirability of this space from the user’s perspective is prioritized over that of the external audience.

Concerning the alterations in housing patterns and the aesthetic and functional prerequisites in densely populated cities such as Tehran, this research aims to develop housing design policy solutions from both an urban observer and the perspective of space users in the realm of semi-open spaces. Residents can retreat from indoor pressures and anxieties on the balcony, which serves as a breather for the interior. Numerous studies have frequently indicated that balconies require both interior and exterior communication. The outcomes can be significantly influenced by the differences in privacy definitions between societies with different cultures and religious beliefs. Therefore, the current study could have resulted in different outcomes for different societies, particularly regarding the perspective from within the space. Furthermore, the interpretation of people’s preferences for architectural designs can be affected by methodological and demographic limitations. The sensory and experiential dimensions of architecture may not be fully captured in artificial lab settings through the use of static images or renderings, methodologically. Age and gender are demographic variables that affect how individuals perceive and evaluate architectural forms. Modern styles may be preferred by younger participants, while older individuals tend to favor traditional aesthetics. An educational background, particularly in design or art, can lead to more critical or nuanced evaluations.

The impact of culture and lifestyle in different cities of Iran, as well as architectural differences over time, and numerous parameters such as presentation of images and respondents’ expertise, considering the dual role of this space, can be reflected in future studies in this field.

## Supporting information

S1 Data Supplementary data. (XLSX)
